# Exosomes derived from gemcitabine-resistant cells transfer malignant phenotypic traits via delivery of miRNA-222-3p

**DOI:** 10.1186/s12943-017-0694-8

**Published:** 2017-07-25

**Authors:** Feng Wei, Chengyuan Ma, Tong Zhou, Xuechao Dong, Qinghua Luo, Li Geng, Lijuan Ding, Yandong Zhang, Li Zhang, Nan Li, Yang Li, Yan Liu

**Affiliations:** 1 Department of Hepatobiliary & Pancreas, the First Hospital of Jilin University, Changchun, Jilin 130021 China; 2 Genetic Engineering Laboratory of PLA, The Eleventh Institute of Academy of Military Medical Sciences of PLA, Changchun, Jilin 130122 China; 3 Department of Respiratory Medicine, the First Hospital of Jilin University, Changchun, Jilin 130021 China; 4 Department of neurosurgery, the First Hospital of Jilin University, Changchun, Jilin 130021 China; 5 Department of Endocrinology, the First Hospital of Jilin University, Changchun, Jilin 130021 China; 6 Department of General Surgery, the Second Hospital of Jilin University, Changchun, Jilin 130041 China; 7 College of Veterinary Medicine, Yangzhou University, Yangzhou, Jiangsu 225009 China

**Keywords:** Exosome, Gemcitabine-resistant, Malignant phenotypic traits, miR-222-3p, Non-small cell lung cancer

## Abstract

**Background:**

Although gemcitabine-based chemotherapy has been established as a core multimodal therapy for non-small cell lung cancer (NSCLC) treatment, its clinical efficacy remains limited by the development of acquired resistance following tumor metastasis and relapse. In this study, we investigated how gemcitabine-resistant (GR) cells contribute to the development of NSCLC tumor malignancy via exosome-mediated transfer of microRNAs.

**Methods:**

We first studied the mechanism of exosome internalization via PKH-67 staining and an immunofluorescence assay, then confirmed our finding by transmission electron microscopy and western blot analysis. Candidate miRNAs were identified through microarray analysis. Thereafter, RT-PCR, MTS, Transwell and soft agar assays were performed to assess the role of exosomic miR-222-3p in vitro. A 3’ untranslated region reporter assay was applied to identify the target of miR-222-3p. A lung metastasis mouse model was constructed to evaluate tumor growth and metastasis in vivo. Finally, clinical samples were used for correlation analysis between exosomic miR-222-3p levels and patients’ response to gemcitabine.

**Results:**

A549-GR–derived exosomes were internalized by receipt cells via caveolin- and lipid raft-dependent endocytosis, which allowed the transfer of miR-222-3p. Exosomic miR-222-3p enhanced the proliferation, gemcitabine resistance, migration, invasion, and anti-anoikis of parental sensitive cells by directly targeting the promoter of SOCS3. In addition, a higher level of exosomic miR-222-3p in sera usually predicted worse prognosis in NSCLC patients.

**Conclusion:**

Our data demonstrate that exosomic-miR-222-3p functions as a principal regulator of gemcitabine resistance and malignant characteristics by targeting SOCS3. The exosomic miR-222-3p level in sera may be a potential prognostic biomarker for predicting gemcitabine sensitivity in NSCLC patients.

**Electronic supplementary material:**

The online version of this article (doi:10.1186/s12943-017-0694-8) contains supplementary material, which is available to authorized users.

## Background

Lung cancer is the leading cause of cancer-related deaths among humans worldwide, and non-small cell lung cancer (NSCLC) accounts for approximately 85% of all lung cancer cases [1]. Even when NSCLC is diagnosed at an operable stage and treatment with chemo- or radio-therapies is applied, the incidence of recurrence and metastasis remains high with a median survival of less than 10–12 months. At present, gemcitabine-based chemotherapy is an established multimodal therapy for NSCLC treatment. However, its clinical efficacy remains limited by the development of acquired resistance following tumor metastasis and relapse. Increasing evidence indicates that micro RNAs (miRNAs) are involved in gemcitabine resistance and tumor progression in several malignant cancers. In breast cancer, miR-21 was found to induce epithelial–mesenchymal transition (EMT) and gemcitabine resistance through activation of the PTEN/AKT pathway [2]. In addition, a recent study found that downregulation of miR-130a-3p can induce the EMT and malignancy of hepatocellular carcinoma cells via inhibition of Smad4 [3]. Also, miR-101-3p reverses gemcitabine resistance by inhibiting ribonucleotide reductase M1 in pancreatic cancer [4].

miR-222 was initially revealed to induce polarization of tumor-associated macrophages in epithelial ovarian cancer [5]. Further studies indicated that elevated levels of miR-222-3p can promote cell proliferation and tumor metastasis via targeting ERα in endometrial carcinoma [6], whereas down-regulation of miR-222 restrains cell proliferation and migration by activating SIRT1 in prostate cancer [7]. Moreover, the miR-221 and miR-222 cluster can regulate TRAIL resistance and enhance tumorigenicity through PTEN and TIMP3 down-regulation [8]. All these findings suggest that miR-222 plays an important role in tumorigenesis and tumor metastasis. However, the precise role of miR-222-3p in modulating malignant progression and chemo-resistance in lung cancer is largely unknown.

As post-transcriptional regulators, miRNAs negatively regulate gene expression by binding directly to the 3’untranslated regions (UTRs) of target mRNAs, thereby inducing mRNA degradation or repression of protein translation. The suppressor of cytokine signaling 3 (SOCS3), a primary member of the SOCS family of proteins, is a negative feedback regulator of the JAK/STAT signaling pathway [9]. Recent studies have revealed SOCS3 overexpression can inhibit cell proliferation and anchorage-independent growth in breast cancer cells [10], whereas down-regulation of SOCS3 reflects a poor prognosis in gastric cancer and hepatocellular carcinoma [11, 12]. However, whether SOCS3 acts as the target of miR-222-3p, thereby contributing to gemcitabine resistance in NSCLC, remains unclear.

Exosomes are nano-sized microvesicles (30–100 nm in diameter) that are formed by the inward budding of late endosomes released into the extracellular environment upon fusion with the plasma membrane [13, 14]. Exosomes can be produced by many cell types, including T cells, B cells, dentritic cells, epithelial cells, and tumor cells [15]. Emerging studies have demonstrated that tumor cells can secrete exosomes into the extracellular space, and the exosomes then migrate far away from their initial position, transferring various types of functional effectors (RNAs, miRNAs, and proteins) to recipient cells [16, 17]. Moreover, studies have shown exosomes play pleiotropic roles in regulating tumor progression, metastasis, chemoresistance and immune dysregulation [18–21]. Several studies have indicated that abundant cell-free miRNAs from exosomes within biological fluids can induce a miRNA-mediated repression of target genes, and these exogenous miRNAs can function effectively in various physiological and pathological processes as either oncogenes or tumor suppressors in recipient normal or tumor cells [22, 23]. Examples include the ability of exosomes derived from hypoxic oral squamous cell carcinoma cells to deliver miR-21 to normoxic cells and elicit a pro-metastatic phenotype as well as the ability of exosomic miR-29a to bind Toll-like receptors in nearby tumor-associated macrophages and trigger a protumoral inflammatory reaction in lung cancer [24]. However, the mechanisms underlying the association of exosomes with gemcitabine resistance during tumor progression and relapse in NSCLC remain poorly understood. Multiple studies have suggested that tumor-derived exosomes can, at least in part, mediate the malignancy of either normal or tumor cells through transported miRNAs, and several exosomal miRNAs now serve as cancer biomarkers and therapeutic targets [25–27]. Given that chemo-resistance can not be induced in all tumor cells simultaneously, we hypothesized that miRNAs might be transported from gemcitabine-resistant (GR) cells to non-resistant cells through exosomes to spread gemcitabine resistance and accelerate tumor progression in NSCLC.

In this study, we evaluated the content of exosomes shed by A549-GR cells and found they are rich in miR-222-3p, which readily entered recipient cells via both caveolin- and lipid raft-dependent endocytosis. We then found that this exosomic transfer of miR-222-3p promoted cell growth and metastasis by down-regulating SOCS3 expression. In addition, our results indicated that the level of exosomic miR-222-3p from patient sera was inversely associated with prognosis and tumor metastasis following gemcitabine therapy. Therefore, our findings suggest a novel molecular mechanism and a potential prognostic biomarker of tumor resistance to gemcitabine in human NSCLC.

## Methods

### Patients and tissue samples

Fifty patients diagnosed with NSCLC were enrolled in the Department of Thoracic Surgery of the First Affiliated Hospital of Jilin University (Changchun, Jilin, China) from January 2012 to December 2014. The clinical and pathologic characteristics of the patients are summarized in Table 1. All patients received routine gemcitabine-platinum chemotherapy (no surgery and completed 2–4 cycles). Informed consent was obtained from each patient, and the protocol was approved by the Ethics Committee of the First Affiliated Hospital of Jilin University.

**Table 1:** Clinicopathologic parameters and circulating exosomal miR-222-3p level in 50 NSCLC patients

Parameters	NO. of Patients	Expression of miR-222-3p^a^		*P*-value
		low	high	
Overall	50	25	25	
Age				
≥ 60	24	14	10	0.396
< 60	26	11	15	
Gender				
Male	40	21	19	0.725
Female	10	4	6	
Histology				
Squamous	22	9	13	0.393
Adenocarcinoma	28	16	12	
Differentiation				
Well	3	1	2	0.820
Moderate	18	10	8	
Poor	29	14	15	
cTNM stage				
II	6	4	2	0.489
III	22	12	10	
IV	22	9	13	
cT stage				
cT1–2	27	16	11	0.256
cT3–4	23	9	14	
cN stage				
cN0	12	9	3	0.095
cN+	38	16	22	
cM stage				
cM0	28	20	8	0.001**
cM1	22	5	17	
Response after Gem^b^				
PR	27	19	8	0.006**
SD	7	3	4	
PD	16	3	13	

^a^Median miR-222-3p level is used as the cut-off

^b^ *PD* Progressive disease, *SD* Stable disease, *PR* Partial response

*P*-value of Fisher’s exact test are shown. **P < 0.01

### Cell culture and transfection

All human NSCLC cell lines were purchased from the Type Culture Collection of the Chinese Academy of Sciences (Shanghai, China) and cultured in Dulbecco’s Modified Eagle Medium (DMEM)/F-12 medium with 10% FBS (Gibco) at 37 °C in 5% CO_2_. The gemcitabine-resistant cell line (A549-GR) was established as rapamycin-resistant cells as described previously [28]. Briefly, gemcitabine-sensitive A549 parental cells (A549-P) were exposed to gradually increasing concentrations of gemcitabine (LC laboratories) from 10 nM initially up to 10 μM over a 6-month period. Transfection of miR-222-3p mimics/inhibitor (Ambion, Carlsbad, CA), or GV-144-SOCS3 plasmid (GeneChem, Shanghai, China) was carried out using Lipofectamine 2000 (Invitrogen) according to the manufacturer’s instructions. Stable A549-P-*luc2*, A549-P-KD-*luc2*, A549-GR-*luc2*, and A549-GR-KD-*luc2* cell lines were established using lentivirus pGC-FU-LUC-IRES-puromycin carrying negative control or oligonucleotides against miR-222-3p (GeneChem), and cells were continually incubated with puromycin (2.5 μg/ml, Sigma) to allow for acquired resistance.

### Exosome isolation

Exosomes were isolated by differential centrifugation of conditioned media collected from A549-P/GR cells. Cells were grown in medium containing 10% exosome-depleted fetal bovine serum (FBS, SBI System Biosciences, Palo Alto, CA, USA). After 3 days’ incubation, the conditioned medium was initially cleared of cellular debris, and the dead cells were removed with two sequential centrifugation steps at 2500 g for 10 min at 4 °C. The supernatants were then spun at 110,000×g for 70 min at 4 °C. The pellets were washed with phosphate-buffered saline (PBS) and the ultracentrifugation protocol was repeated. The final exosome pellet was resuspended in PBS. To isolate exosomes from human peripheral blood, samples were centrifuged twice at 2000×g for 10 min to separate the plasma from red blood cells, and exosomes were isolated via ultracentrifugation as described above. Protein amounts in exosomes were quantified using the bicinchoninic acid assay.

### Transmission electron microscopy (TEM)

First 20 μg of exosomes was loaded onto parafilm, and then a 300 mesh copper grid (Agar Scientific Ltd., Stansted, UK) was placed over the drop for 2 min. After the excess liquid was removed by blotting with filter paper, the grid was negatively stained with 2% phosphotungstic acid (PTA) for 2 min and examined at 80 kV with a JEM-1200 EXII TEM (JEOL, Ltd., Tokyo, Japan).

### Immunofluorescence assay

Purified exosomes were labeled with green fluorescent linker PKH-67 (Sigma) according to the manufacture’s protocol. Cells were seeded in 8-well chamber slides (8000 cells/well) and pre-treated with pharmacological inhibitors for 2 h. Then 5 μl of PKH67-dyed exosomes were added before a 4-h incubation to allow internalization. Finally, slides were washed twice with PBS, fixed with 4% paraformaldehyde (PFA), and mounted with DAPI-containing mounting media (Vector Labs). Images were taken using a Zeiss LSM 780 (Zeiss, Jena, Germany) confocal microscope.

### Western blot analysis

Total proteins were extracted using an extraction buffer with a protease inhibitor cocktail (Thermo Scientific), and equal amounts of protein (50 μg) were separated by sodium dodecyl sulfate (SDS)-polyacrylamide gel electrophoresis and transferred to polyvinylidene fluoride (PVDF) membranes (Millipore). Membranes were blocked and probed with primary antibodies overnight including those against Alix, TSG101, CD81, SOCS3, JAK2(T/P), Stat3(T/P), Bcl-2, Bax and Bcl-XL (Cell Signaling Technology and Abcam). After incubation with secondary antibodies, the membranes were developed for chemiluminescence measurement.

### Quantitative real-time polymerase chain reaction (PCR)

Total RNA was isolated from cells or exosomes using RNeasy Kit (Qiagen). cDNA was synthesized from 1 to 10 μg RNA using the TaqMan® MicroRNA Reverse Transcription Kit (Applied Biosystems). Aliquots of the reaction mixture were used for PCR with TaqMan® 2× Universal PCR Master Mix. All PCR experiments were performed in triplicate. The U6 RNA level was used as an internal control for data normalization.

### Cell proliferation assay

Cells were seeded in 96-well plates at 8000 cells/well and cultured overnight. After treatment with exosomes or drugs for 48 h, MTS was added at 20 μl/100 μl medium. The absorbance was measured with a spectrophotometer (Bio-Rad Inc) at 490 nm, and cell growth inhibition was calculated using the equation: cell viability (%) = (At/Ac) × 100%, where At and Ac represent the absorbance in the treated and control cultures, respectively [12].

### Colony formation assay

Cells were trypsinized (single-cell suspension) and seeded into 6-well plates (800 cells/well) for culture in medium containing gemcitabine (1 μM), which was refreshed every 3 days. After 10 days of treatment, the medium was discarded, the cell colonies were stained with crystal violet (0.1% in 20% methanol) and imaged using a digital camera to record the results.

### Luciferase reporter assay

The dual-luciferase vectors psiCHECK-SOCS3–3’UTR-WT and psiCHECK-rcmiR −222-WT were constructed by synthesizing the candidate seed sequences in the 3′-UTR of SOCS3 or the reverse complementary sequence of miR-222 and inserting them into the psiCHECK-2 vector. For mutant vectors, 3–4-bp mutations were introduced into the seed sequences. All plasmids were confirmed by DNA sequencing. For reporter assays, HEK-293 T cells were seeded in 24-well plates and transfected with 0.8 μg recombinant vectors alone or plus 30 nM mimics or inhibitors. Firefly and Renilla luciferase activities in cell lysates were measured 48 h later using the dual-Glo reporter assay system (Promega).

### Cell migration assay

A wound-healing assay was performed to assess any influence of treatment on cell migration. Cells were seeded in 6-well plates to create a confluent monolayer, and then a scratch wound was made with a sterile pipette tip in a straight line. After rinsing with PBS, cells were incubated with medium containing exosomes (10 μg/well, 0.5% FBS). Images were captured under a microscope at 12 h or 24 h post-wounding.

### Cell invasion assay

An invasion assay was performed using 6.5-mm Transwell chambers with 8-μm pores (Costar) according to the manufacturer’s instructions. Briefly, 1 × 10^5^ cells in serum-free medium were seeded in the upper insert precoated with matrigel (1:4, BD Biosciences), and then 600 μl complete medium containing 10 μg exosomes was added to the bottom chamber as a chemoattractant. After incubation for 24 h, the upper surface of each membrane was cleaned, and cells adhered to the insert surface were fixed and stained with 0.5% crystal violet.

### Anchorage-independent soft agar assay

Cells were suspended in 0.35% agarose and medium supplemented with 10% FBS, and the mixture was seeded in 6-well plates containing a basal layer of 0.6% agarose at 5000 cells/well. Then medium was replaced twice per week. After 3–4 weeks of routine culture, colonies were stained with 0.005% crystal violet for longer than 1 h, and images were captured using an SZX12 microscope (Olympus, Japan). Viable colonies larger than 0.1 mm in diameter were counted.

### Animal experiments

Animal experiments were performed according to protocols approved by the Institutional Animal Care and Use Committees of Jilin University. Female severe combined immunodeficient (SCID) mice at ~6 weeks old (Vital River, Beijing, China) were injected 1 × 10^6^ viable tumor cells via the tail vein. The mice were treated with PBS or exosomes (*n* = 8 each). Successful injections were confirmed by immediate luciferase imaging. For this, mice were anesthetized and injected intraperitoneally with luciferin (25 mg/ml in 100 μl PBS), and images were collected beyond 15 min post-injection using an IVIS-Lumina system (Caliper, USA). Light emission from animal tissue (photons/s) was measured using software provided by the vendor (Xenogen, Corp., Alameda, CA). Luciferase imaging was performed once a week for a total of 6 weeks unless significant morbidity and earlier euthanasia were required. Finally, mice were sacrificed, and tumor tissues were frozen in liquid nitrogen.

### Statistical analysis

All statistical analyses were performed using GraphPad Prism software (San Diego, CA). Data are presented as mean value ± standard error, and the clinicopathological parameters were compared using the Fisher’s exact test. The statistical significance of differences between two groups was analyzed using two-tailed unpaired Student’s t-tests, and *P* < 0.05 was considered to be statistically significant.

## Results

### A549-GR cell-derived exosome (GR-Exo) internalization is mediated through caveolin- and lipid raft-dependent endocytosis

**Fig. 1:**
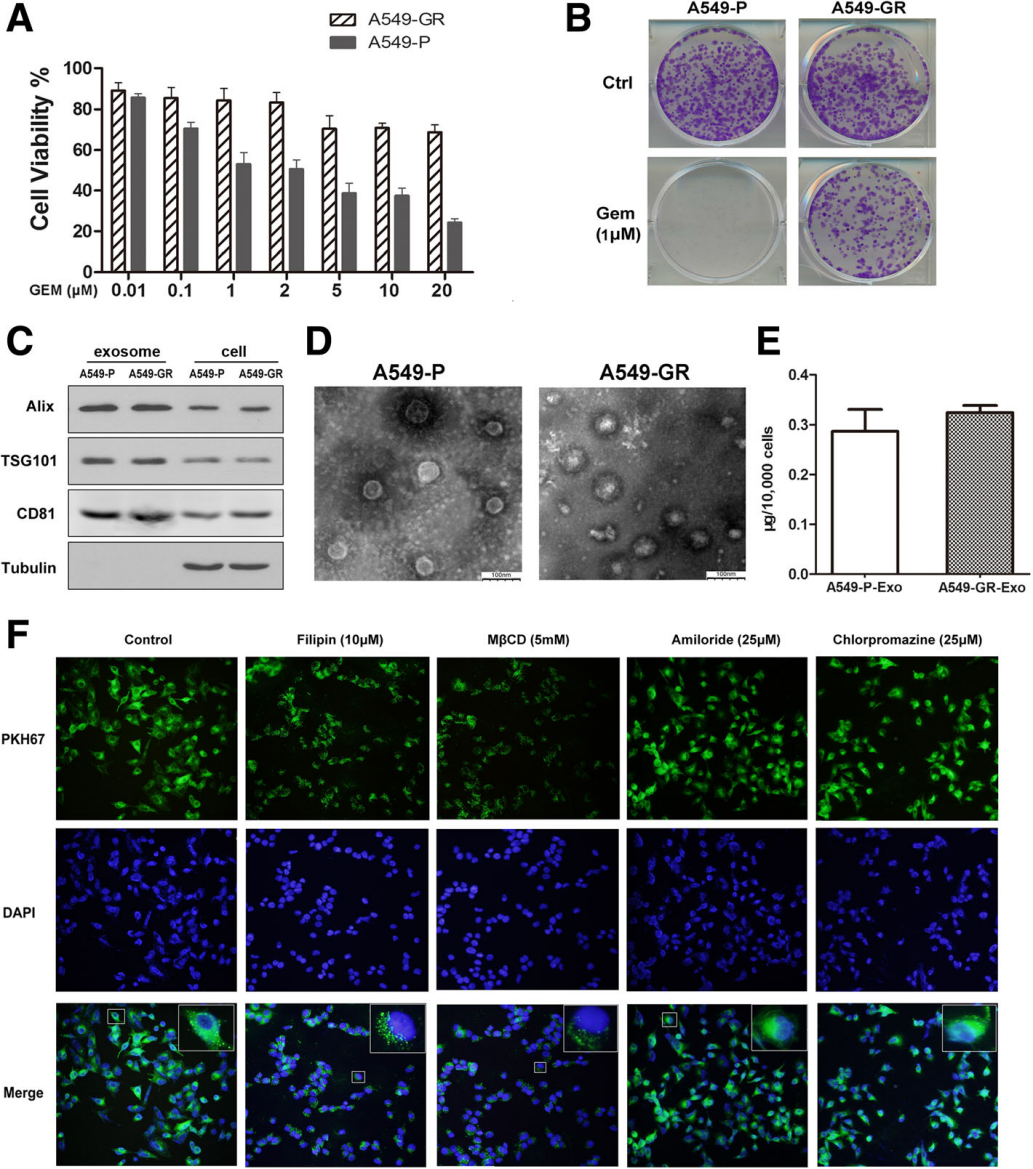
Gemcitabine-resistant cells shed exosomes that readily enter A549-P cells. **a** A549-P/GR cells were treated with increasing concentrations of gemcitabine for 72 h. Cell growth was analyzed by MTS assay. Data represent at least three experiments performed in triplicate. Error bars represent ± SD. *P < 0.01; **P < 0.001. **b** A549-P/GR cells were treated with gemcitabine (1 μM) and examined by colony formation assay. **c** The presence of TSG101, CD81, and Alix in 15 μg of exosomes was analyzed by western blot. **d** Exosomes were observed by transmission electron microscopy. Scale bar, 100 μm. **e** Quantification of exosomes expelled from equal numbers of A549-P/GR cells by BCA analysis. **f** A549-P cells were treated with different pharmacological inhibitors of endocytic pathways for 2 h. A549-GR–derived exosomes (GR-Exo) were dyed with PKH67 (green) and incubated with pretreated cells for 4 h, and exosome uptake was viewed under confocal microscopy

To identify the differences in characteristics between A549-GR and A549-P cells, we first measured the cell viability of each line after exposure to different concentrations of gemcitabine. The MTS colorimetric assay demonstrated that A549-GR cells were more resistant than A549-P cells to increasing gemcitabine concentrations (0.01–20 μM; Fig. 1a). Also, similar results were obtained in the colony formation assay (2.5 μM gemcitabine; Fig. 1b), indicating that A549-GR cells have greater chemoresistance capability to gemcitabine treatment.

We then isolated the exosomes from the conditioned medium of A549-P and A549-GR cells. Western blot analysis demonstrated the expression of TSG101, CD81, and PDC6I/Alix, which are all exosome markers and associated with exosome formation, in both exosomes and cells (Fig. 1c). In addition, TEM revealed that the predominant vesicles were of typical exosomal size (30–100 nm in diameter) with the characteristic round or “saucer shape” delineated by a lipid bilayer (Fig. 1d). Further quantitative analysis suggested that the amount of exosomes expelled from both cell variants did not differ significantly (*P* > 0.05; Fig. 1e).

To clarify the mechanisms of GR-Exo entry in NSCLC cells, pharmacological inhibitors of various endocytic pathways were used to suppress exosome internalization in A549-P recipient cells. As indicated in Fig. 1f, exosome internalization was remarkably inhibited upon treatment with 10 μM filipin (caveolin inhibitor) or 5 mM MβCD (lipid-raft inhibitor). Quantification of internalized exosomes (green) showed a 45.5% reduction with 10 μM filipin treatment and a 57.5% reduction with 5 mM MβCD treatment. In addition, treatment with amiloride (macropinocytosis inhibitor) or chlorpromazine (clathrin inhibitor) did not show significant inhibition of exosome internalization (6.5% *or* 5.8%), even at concentrations as high as 100 μM and 50 μM, respectively. These results indicated that exosomes were internalized by NSCLC cells mainly through both caveolin- and lipid raft-dependent endocytosis, but not macropinocytosis- and clathrin-dependent endocytosis.

### GR-Exo transfer miR-222-3p to recipient cells and confer malignant phenotypes

**Fig. 2:**
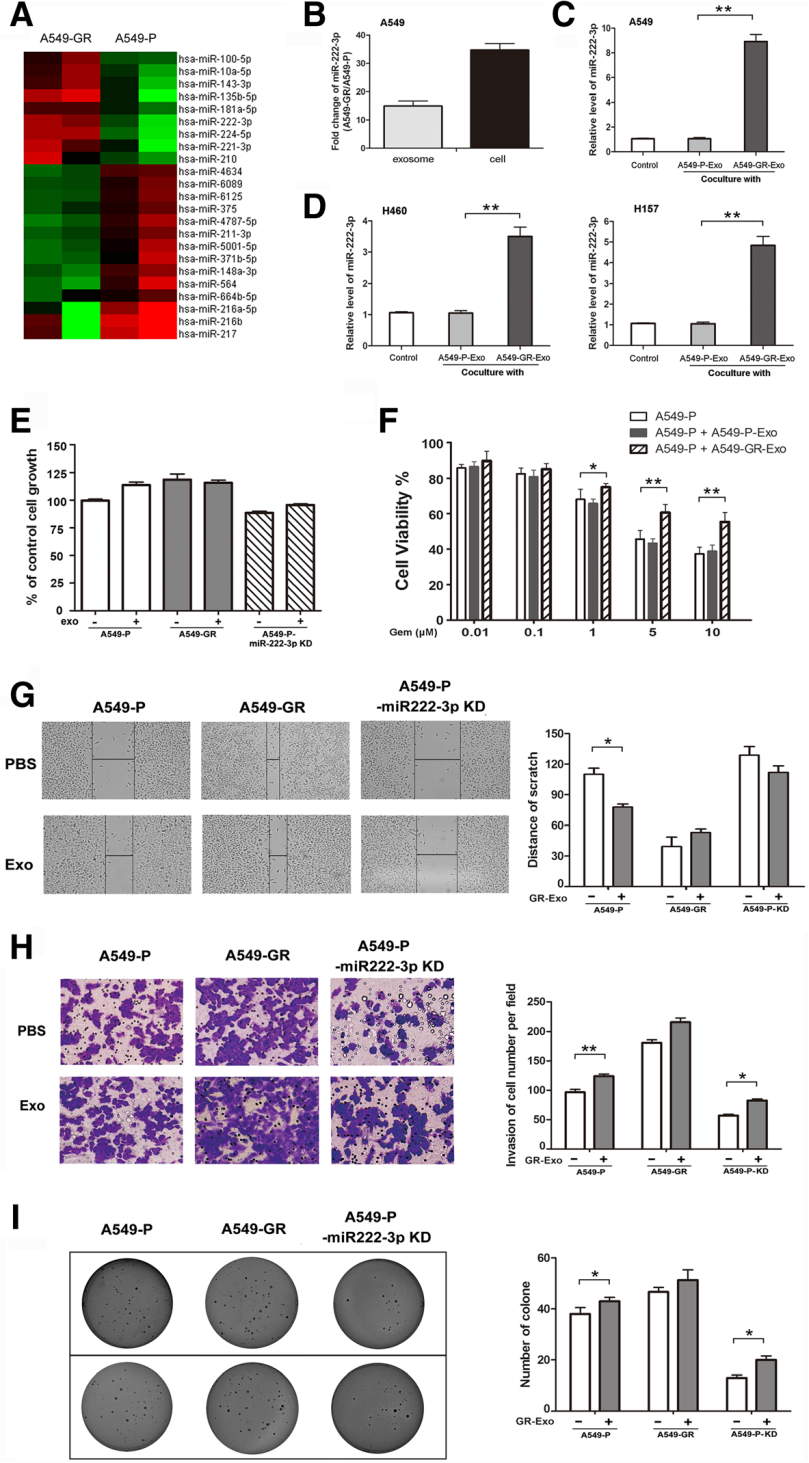
GR-Exo deliver miRNA-222-3p into recipient cells, enhance gemcitabine resistance, promote cell metastasis and proliferation. **a** Heatmap of differential miRNA expression between A549-GR and A549-P exosomes. Gene expression data were obtained using a human microRNA array. Expression values shown are mean centered. Red: increased expression, Green: decreased expression. **b** miR-222-3p expression was detected in A549-P/GR cells and A549-P/GR–derived exosomes by qRT-PCR. **c** miR-222-3p expression was detected in A549-P cells after co-incubation with P-Exo or GR-Exo for 24 h by qRT-PCR. **d** miR-222-3p expression was detected in H460 and H157 cells after co-incubation with P-Exo or GR-Exo by qRT-PCR. **e** The growth rates of A549-P and A549-P-KD cells in the absence or presence of GR-Exo were assessed using MTS assay. **f** Cell growth was assessed by MTS after treatment with gemcitabine for 72 h in the absence or presence of P-Exo or GR-Exo. **g** The migration of exosome-treated cells was assessed using wound-healing assay. Right, quantitative analysis of invasive cells. **h** Transwell assay was performed to assess cell invasion. Right, quantitative analysis of scratch wound closure. **i** Soft agar assay was performed to examine the anchorage-independent survival of cells. Right, quantitative analysis of cell clones. Data represent at least three experiments performed in triplicate. *P < 0.05; **P < 0.01

Noting many miRNAs are involved in tumor cell-derived exosomes [29], we expected that GR-Exo might transfer some crucial miRNAs to recipient cells to promote malignancy in NSCLC cells. Our miRNA array data demonstrated that the level of miR-222-3p was upregulated by 16.7-fold in GR-Exo compared to that in P-Exo (Fig. 2a). Similarly, a parallel examination indicated that the miR-222-3p expression was 37.01-fold higher in donor cells (A549-GR/A549-P) from which the exosomes were derived (*P* < 0.05, Fig. 2b). Importantly, we noticed that GR-Exo can induce the upregulation of miR-222-3p, whereas A549-P-Exo failed to have similar effect in recipient A549-P cells (Fig. 2c). Moreover, we also found that GR-Exo displayed similar effects in H460 or H157 cells (Fig. 2d). All these results suggest that exosomes might deliver miR-222-3p from GR donor cells to gemcitabine-sensitive recipient cells.

We next investigated the effects of GR-Exo on the proliferation and gemcitabine resistance of sensitive A549-P cells. Our results indicated that the cell morphology did not exhibit obvious alteration after treatment with exosomes for 48 h, and a moderate increase in the proliferation of A549-P cells was detected in the presence of exosomes (1.14 ± 0.05, *P* < 0.05), which better matched the growth rate of A549-GR cells (Fig. 2e, left). Moreover, the exosomes induced a certain gemcitabine resistance in A549-P cells in a dose-dependent manner (Fig. 2f). Notably, the exosomes failed to promote cell proliferation and gemcitabine resistance in miR-222-3p knockdown (KD) A549-P cells, and miR-222-3p KD further decreased cell proliferation and enhances cell sensitivity to gemcitabine (Fig. 2e, right panel), suggesting that miR-222-3p might be involved in this process.

We further evaluated the effects of GR-Exo on cell migration and invasion of sensitive A549-P cells. Figure 2g and h showed that the exosomes induced a significantly increased wound closure and invasion capability. As expected, the effects of GR-Exo could be significantly inhibited in miR-222-3p-KD A549 cells in vitro, indicating that miR-222-3p plays important roles in the effects of GR-Exo on cell migration and invasion of sensitive A549-P cells. Noting that drug-resistant cells often confer anti-anoikis to promote tumor recurrence and metastasis, an anchorage-independent soft agar assay was employed to confirm the exosomes could induce increased cell survival under anchorage-independent conditions, and miR-222-3p knockdown could suppress cell survival in soft agar. Furthermore, before the start of above experiments, we evaluated the level of miR-222-3p to ensure the alteration of cell malignance could attribute to miR-222-3p. Our results indicated that the level of miR-222-3p was significantly upregulated after GR-Exo treatment, however, miR-222-3p was potently suppressed in miR-222-3p-KD A549 cells either with or without GR-Exo treatment Additional file 1: Figure S1). Collectively, these results demonstrated that GR-Exo could promote drug resistance, migration, invasion, and anchorage-independent survival of sensitive NSCLC cells, and distinctly, miR-222-3p works as a crucial effector (Fig. 2i).

### Exosomic miR-222-3p inhibits SOCS3 expression by directly targeting its 3′-UTR

**Fig. 3:**
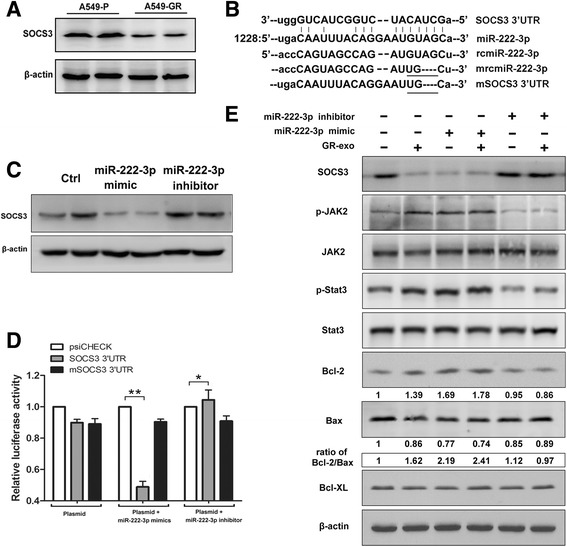
SOCS3 is the direct target of miR-222-3p. **a** SOCS3 expression was examined by western blotting. **b** Sequence alignment of miR-222-3p with reverse complementary miR-222-3p (rcmiR-222-3p), SOCS3 (SOCS3 3’UTR), mutant rcmiR-222-3p (mrcmiR-222-3p), and mutant SOCS3 (mSOCS3 3’UTR). **c** SOCS3 expression in A549-P cells after transfection with miR-222-3p mimic or inhibitor was examined by western blotting. **d** The promoter activities of SOCS3 were measured using the dual-Luciferase reporter assay after transfection of A549-P cells alone or in the presence of miR-222-3p mimic or inhibitor. Data are representative of three experiments. Error bars represent ±SD. *P < 0.05; **P < 0.01 vs. vector alone group. **e** Expression levels of SOCS3/JAK2/Stat3 and Bcl-2/Bax after transfection with miR-222-3p mimic and inhibitor were assessed by western blotting

Given that miRNAs exert biological functions by targeting their specific genes, prediction algorithms TargetScan and MicroRNA were applied to predict the target genes of miR-222-3p. Suppressor of cytokine signaling 3 (SOCS3), a crucial negative regulator of the JAK/STAT signaling pathway, which might be associated with tumor malignant phenotypes [12, 30, 31], was identified as a potential target gene of miR-222-3p. Further determination indicated that SOCS3 expression was inhibited in A549-GR cells compared with A549-P cells (Fig. 3a), which was inversely correlated with the miR-222-3p level in these two cell lines. In addition, we found that miR-222-3p mimic dramatically suppress SOCS3 expression, whereas treatment with an miR-222-3p inhibitor could increase SOCS3 expression in A549-P cells (Fig. 3b). Furthermore, the dual-luciferase reporter assay indicated that miR-222-3p apparently repressed, whereas inhibitor upregulated, Rluc activity involving the seed sequence in the 3’-UTR of SOCS3 in HEK293T cells transfected alone or co-transfected with miR-222-3p mimic/inhibitor, whereas treatment with a mutant of the SOCS3 3’-UTR binding site abolished this effect, suggesting that miR-222-3p inhibits SOCS3 expression by directly targeting its 3’-UTR (Fig. 3c and d).

Furthermore, we confirmed that miR-222-3p mimic could decrease the expression of SOCS3 in recipient A549-P cells, followed by increased activation of p-Jak2 and p-Stat3 as well as an increased ratio of Bcl-2/Bax with or without exosome treatment. Conversely, miR-222-3p inhibitor induced the opposite effects in this process (Fig. 3e). The similar alteration of SOCS3 was observed in A549-GR cells after treated with miR-222-3p mimic/inhibitor or GR-Exo (Additional file 1: Figure S2). All these results suggest that SOCS3 is a direct target of miR-222-3p, and exosomic miR-222-3p might enhance tumor malignancy through SOCS3 and its downstream effectors, such as the Jak2/Stat3 and Bcl-2 pathways.

### Exogenous overexpression of SOCS3 abolishes GR-Exo–induced tumor malignancy in NSCLC cells

**Fig. 4:**
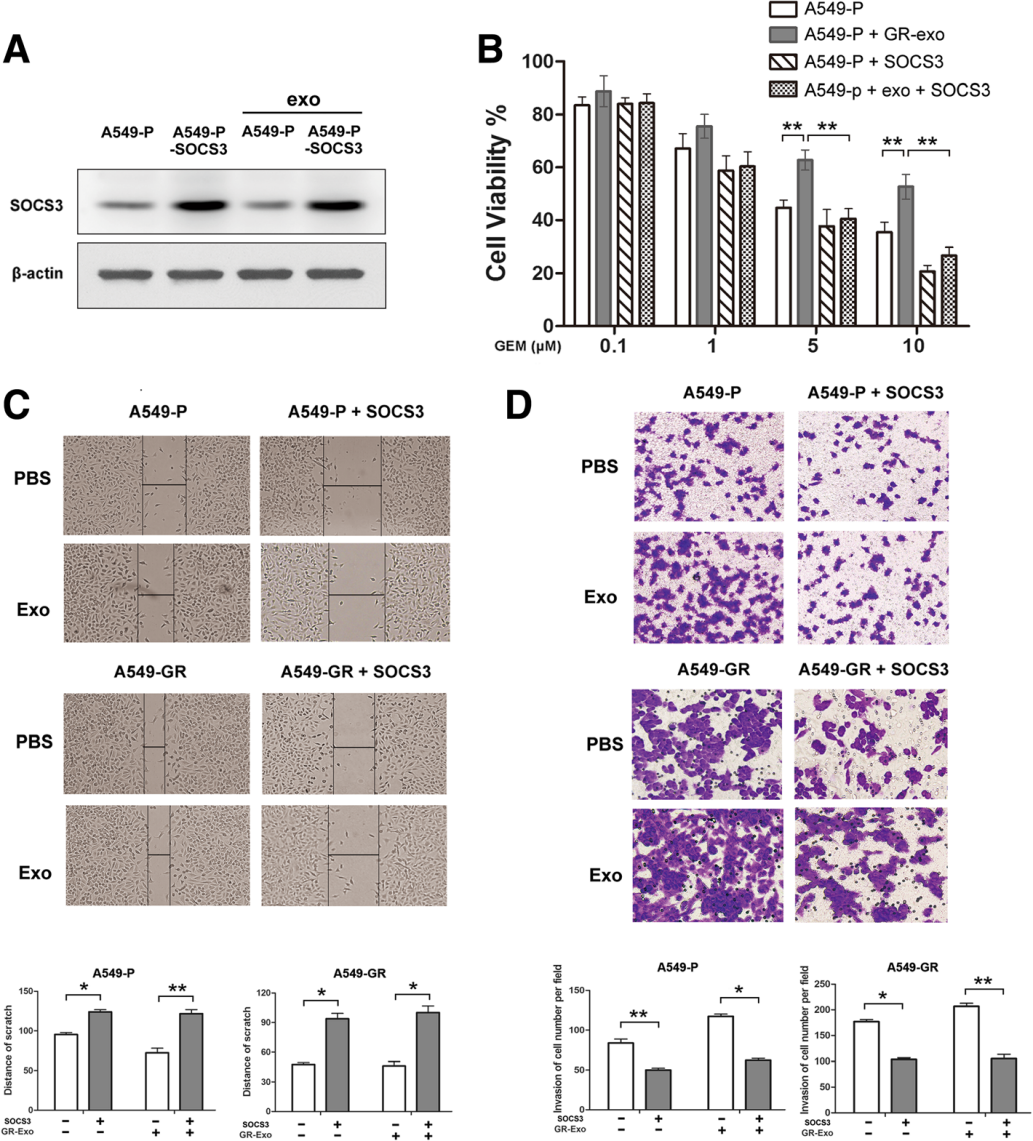
Exogenous overexpression of SOCS3 rescues A549-P cells from GR-Exo–induced stronger malignancy. A549-P or A549-GR cells were transfected with GV-144-SOCS3 plasmid and treated with GR-Exo. **a** SOCS3 expression was assessed by western blotting. **b** Cell viability was assessed by MTS after co-treatment with gemcitabine for 72 h. Data are representative of three experiments. Error bars represent ±SD. *P < 0.05; **P < 0.01 vs. control. **c** Wound-healing assay and **d** Transwell assay were performed to assess cell migration and invasion. Lower, quantitative analysis of scratch wound closure (**c**) or cell invasion (**d**). Data represented at least three experiments performed in triplicate. *P < 0.05; **P < 0.01

According to our results described above, it was reasonable to infer that up-regulation of SOCS3 expression might reverse the malignant traits that were aggravated by GR-Exo–transferred miR-222-3p. As shown in Fig. 4a, exogenous SOCS3 transfection effectively increased the expression of SOCS3, either with or without GR-Exo treatment. Moreover, overexpression of SOCS3 alleviated GR-Exo–induced malignant phenotypes, with effects such as inhibiting cell proliferation, sensitizing A549-P cells to gemcitabine, and inhibiting cell migration and invasion (Fig. 4b-d). Because overexpression of SOCS3 could not only restore but alleviate the malignant characteristics of sensitive cells beyond the original level, we expected that the effects of SOCS3 overexpression might play even more important roles in tumor malignancy than miR-222-3p and the other plentiful tumor-related proteins and miRNAs from GR-Exo.

### Knockdown of miR-222-3p inhibits lung metastasis of NSCLC in vivo

**Fig. 5:**
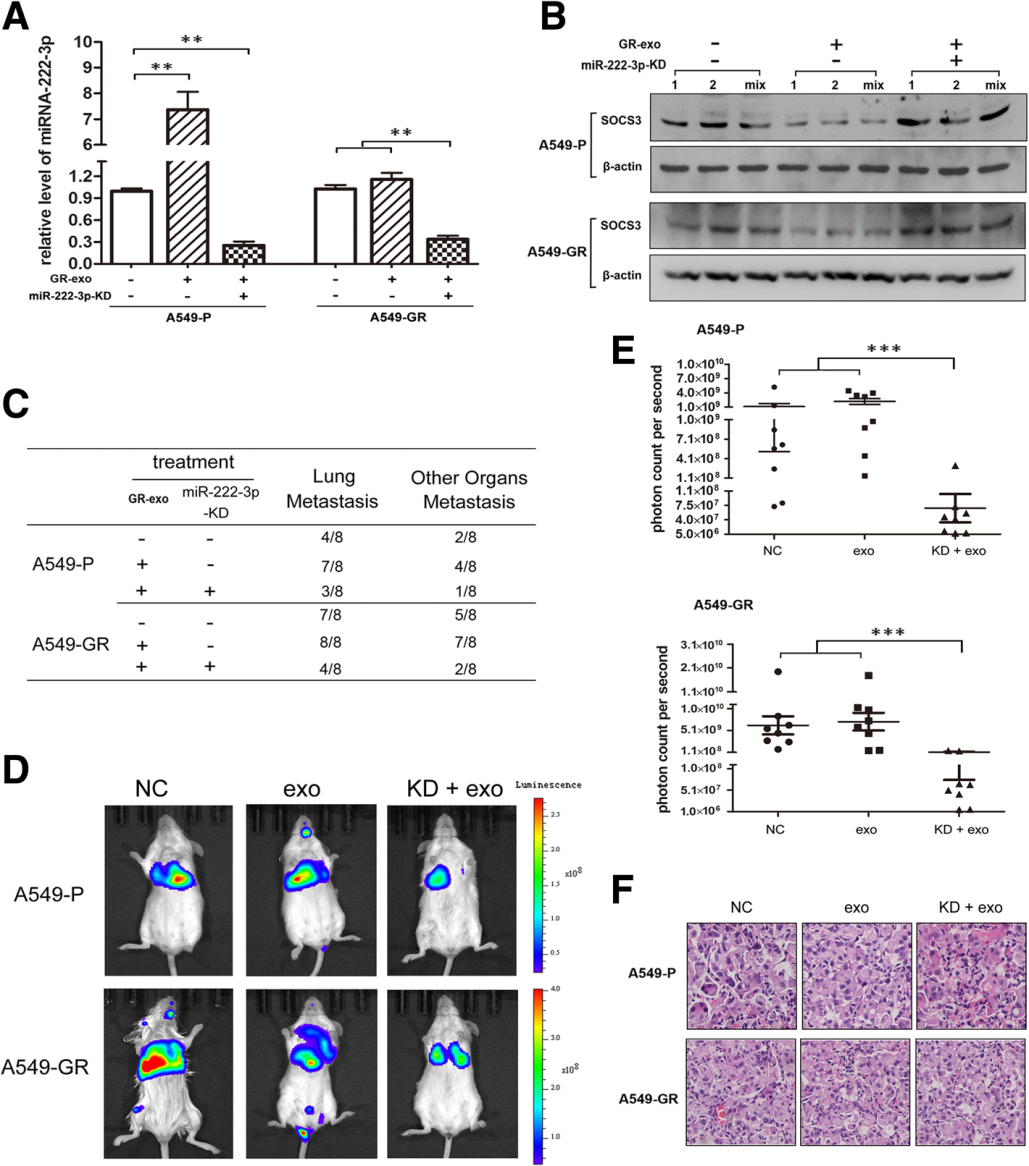
Knockdown of miR-222-3p in A549-P/GR cells suppresses tumor growth and lung metastasis in vivo*.* Lung metastasis models in SCID mice were generated by using luciferase-labeled A549-P/GR cells with lentivirus miR-222-3p KD or normal control (NC) and treated with PBS (control) or GR-Exo (*n* = 8). **a** miR-222-3p expression was assessed by qRT-PCR. **b** SOCS3 expression was assessed by western blotting. Two samples were selected from each group randomly (*n* = 8) (labeled as 1,2), and all samples were sufficiently mixed as a pool (labeled as mix). **c** The incidence of metastasis in the lung or other organs in each group. **d** Luminescence was assessed in 8 mice of each group at 6 weeks after injection. **e** To quantify the metastatic mass, the photon counts per second were recorded (***P < 0.001). **f** The lungs of mice from each group were removed, and lung metastasis was evaluated by hematoxylin and eosin (H&E) staining

To further investigate the potential effects of exosomic miR-222-3p in vivo, we generated a lung metastasis model via tail vein injection of SCID mice. As shown in Fig. 5a, GR-Exo induced a significant increase of miR-222-3p in the A549-P group, and a minor increase in the A549-GR group, whereas GR-Exo failed to induce this increase in miR-222-3p in the A549-P-KD and A549-GR-KD groups. The expression of SOCS3 was consistent with the miR-222-3p level in each group (Fig. 5b). Moreover, in mice that received A549-GR-*luc2* cells, visible lung metastases were observed in seven mice and later 8 mice after exosome treatment (*n* = 8). We also noticed that GR-Exo increased tumor metastases to other organs, including brain, liver, and bone from 62.5% (5/8) to 87.5% (7/8). Importantly, miR-222-3p KD effectively restricted tumor metastasis to 50% (4/8) for lung metastases and 25% (2/8) for other organs metastases after GR-Exo treatment. Similar results were obtained in mice that received A549-P-*luc2* cells, with GR-Exo promoting both lung metastases (from 50% to 87.5%) and other organ metastases (from 25% to 50%), whereas miR-222-3p KD significantly inhibited tumor metastases (37.5% with lung metastases and 12.5% with other organ metastases; Fig. 5c and d). Moreover, the bioluminescence assay demonstrated metastases in each mouse in all six groups, which is also consistent with our in vitro results showing that GR-Exo enhanced tumor metastasis and this effect was stronger in the A549-P group than in the A549-GR group. Moreover, knockdown of miR-222-3p efficiently repressed metastasis in both the A549-P and A549-GR groups in the presence of GR-Exo (Fig. 5e). Histological analysis of the lungs revealed metastatic tumors in mice that received cells and treatment, and no significant difference was observed (Fig. 5f). Considering that the effect of miR-222-3p KD in the presence of GR-Exo could reflect its potential better than that in the absence of GR-Exo, we saved the mice group designated for miR-222-3p KD and no GR-Exo treatment. Taken together, these data demonstrate that miR-222-3p KD effectively reduced tumor growth and metastasis of NSCLC even with GR-Exo in vivo.

### Circulating exosomal miR-222-3p levels correlate with tumor metastasis and poor prognosis following gemcitabine therapy in NSCLC patients

To evaluate the correlation between exosomic miR-222-3p and the clinic pathologic parameters and response after gemcitabine therapy in 50 NSCLC patients, we isolated the exosomes from the sera of NSCLC patients. The quantity of exosomes in sera (about 330–420 μg/200 μl) did not differ significantly among the patients. The median miR-222-3p level was regarded as the cut-off point for low and high expression (0.007445, which was determined by qRT-PCR with normalization to U6 RNA as an endogenous control), and the circulating exosomic miR-222-3p level was closely associated with tumor metastasis in NSCLC patients (*p* = 0.001) (Table 1). Interestingly, the exosomic miR-222-3p level was significantly correlated with the patient response to gemcitabine treatment (*p* = 0.006), a high level of miR-222-3p usually predicted a negative response to gemcitabine including progressive disease (PD), whereas a low level of miR-222-3p was associated with a positive response including partial response (PR). These results indicate that NSCLC cells that acquired gemcitabine resistance produced exosomes carrying greater amounts of miR-222-3p, and these exosomes could transfer and promote gemcitabine resistance in sensitive cells. These events could also be responsible for the malignant phenotypes and be used to predict worse prognosis after gemcitabine-based chemotherapy in patients with NSCLC.

## Discussion

Gemcitabine is one of the most important chemotherapeutic drugs for human NSCLC treatment, and it improves survival through its abilities to inhibit DNA chain termination, cell cycle progression, apoptosis and metastasis [32, 33]. Especially for elderly patients with advanced NSCLC, a mono-chemotherapy treatment with gemcitabine or docetaxel is the recommended option, because it offers survival and quality of life benefits with acceptable toxicity [34]. However, side effects and acquired resistance still limit the clinical efficacy of gemcitabine therapy. Previous studies indicated that several resistance-related proteins, such as P-glycoprotein, topoisomerases, and thymidylate synthase, are involved in this process [35]. Herein, we aimed to clarify the effects of exosomes from GR cancer cells on other sensitive cells as well as their effects on cancer cell proliferation, tumor metastasis, and chemoresistance in NSCLC treatment.

It is well recognized that several miRNAs are involved in tumor resistance to gemcitabine-based chemotherapy. Dhayat et al. demonstrated miR-138, miR-147b and miR-99a are upregulated, and miR-31 and miR-422a are downregulated in PANC-1-GR cells when compared with parental/sensitive cells [36]. Furthermore, emerging evidence indicates that tumor-derived exosomes as vesicular carriers frequently transfer miRNAs into subcellular sites in recipient cells to induce repression of the expression of certain target genes [23, 37]. However, fewer studies have focused on the roles of GR cell-derived exosomes in onco-miRNA transfer to sensitive cells, and their subsequent roles in the malignancy of receipt cells during NSCLC progression.

In this study, we first purified nano-sized exosomes that were abundantly present in tumor cell-conditioned media of both GR and parental NSCLC cells. Our results indicated that the internalization of A549-GR–Exo was mainly mediated via caveolin- and lipid raft-dependent endocytosis mechanisms, rather than micropinocytosis- or clathrin-dependent endocytosis (Fig. 1). Intriguingly, this is not completely consistent with its effects seen in other cells lines. Pancreatic cancer-derived exosomes can enter paraneoplastic β-cells through caveolin- mediated endocytosis or micropinocytosis [38], and internalization of glioblastoma cell- derived exosomes was shown to be mediated by lipid raft-dependent endocytosis, suggesting that exosome internalization might be closely associated with cell phenotypes, which are mediated by different protein distributions or signaling activations on the cell membrane [39].

In the present study, to elucidate the functions of exosomic miRNAs in gemcitabine resistance and tumor malignancy, a microRNA array was performed to identify 116 miRNAs that were differentially expressed between A549-GR and A549-P exosomes, including 45 upregulated and 70 downregulated miRNAs. Among these differentially regulated miRNAs, 23 miRNAs were found to be significantly up- or down- regulated by at least 5-fold between these two cell lines (*P* < 0.01). Given the conflicting reports regarding whether exosomal miRNA profiles resemble those of parental cells [40, 41], we compared the most remarkably upregulated miRNAs. miR-222-3p was upregulated in A549-GR exosomes to a level even higher than in donor A549-GR cells and could be internalized by receipt cells. We also found the level of miR-222-3p was elevated from A549-P to A549-GR cells, indicating that miR-222-3p might play an important role in tumorigenesis and progression (Fig. 2). Additionally, we observed some other miRNAs were altered in A549-GR-Exo or recipient cells, such as miR-135b, miR-10a, and miR-221-3p were upregulated in both exosomes and recipient cells, whereas miR-181a was specially increased in exosomes, miR-224-5p was upregulated in GR cells and exosomes, but failed to transfer to recipient cells effectively. These findings suggest the exosomic miRNA profile does not resemble that of donor or recipient cells completely. We expect that some miRNAs may not be suitable for packaging into exosomes, and some miRNAs may be less stable in the cytoplasm than in exosomes and tend to degrade.

Although miR-222 has been demonstrated to be overexpressed in several types of cancer and to promote tumor progression and drug resistance via genetic or epigenetic mechanisms [8, 42, 43], the functions of exosomic miR-222-3p and the molecular mechanisms by which miR-222-3p modulates drug resistance and tumor progression have yet to be revealed. Our data demonstrate that miR-222-3p KD in recipient cells could inhibit gemcitabine resistance, colony formation, tumor invasion, and migration in vivo and in vitro (Figs. 2 and 5). Further investigation revealed that miR-222-3p block the expression of SOCS3 by directly binding with the 3’-UTR of SOCS3 to upregulate the expression levels of Stat3 and Bcl-2 (Fig. 3). Constitutive activation of Stat3 had been confirmed to inhibit apoptosis and promote migration in various tumors, including lung cancer [44]. Moreover, we also clarified that the exogenous expression of SOCS3 neutralized the activity of SOCS3 and alleviated GR-Exo–induced malignancy, subsequently (Fig. 4). Bcl-2 families are key regulators of cell anti-apoptosis, the increased ratio of Bcl-2/Bax better elucidates Bcl-2, acts as an downstream effector of SOCS3, contributes to miR-222-3p induced cell anti-apoptotic (Fig. 3e). Additionally, we observed Bcl-XL (anti-apoptotic protein) has a slight decrease, our explanation is that some other mechanisms might be involved in regulating the Bcl-XL expression, especially, when cells received so many exosomic-miRNAs simultaneously, and this data suggest that Bcl-2, but not Bcl-XL plays key roles in inducing cellular anti-apoptosis in cells which involving increased miR-222-3p. Regretfully, our current study could not explain why gemcitabine induced the upregulation of miR-222-3p in NSCLC, although some studies reported that nuclear factor (NF)-κB and c-Jun can induce the expression of miR-221&222 in prostate carcinoma and glioblastoma cells. In addition, over-activated TEAD1 upregulates miR-222 expression via physically binding to its promoter in gastric cancer cells, and a DNA damaging agent can cause dysregulation of miRNA expression at the transcriptional level [45, 46].

Additionally, our results demonstrated that A549-P–derived exosomes failed to promote malignant phenotypes of A549-P cells, and GR-Exo had no strong influence on the proliferation of receipt A549-P cells, which is not consistent with the previous finding that tumor-secreted exosomes derived from BT-474 cells can accelerate the proliferation of BT-474 cells [47, 48]. With increasing evidence that tumor-derived exosomes can confer either anti-tumorigenic or pro-tumorigenic effects [49], one probable explanation for these seemingly conflicting effects may relate to the complex interactions between exosomes, recipient cells, and intercellular environmental factors [50]. On the other hand, the original proliferation rate of A549-P cells is very fast, so it would be difficult to obtain the further promotion. Meanwhile, our data reveals even A549-GR cells involving high level of miR-222-3p, the proliferation rate only increases less than 20% when compared with parental cells (Fig.2e), which is helpful to explain why A549-GR-derived exosomes show only marginal survival benefit. Importantly, this data is not contradicted with our conclusion that GR-Exo indeed deliver the functional miR-222-3p into recipient cells, and certainly, the role of miR-222-3p in enhancing the drug-resistance and motility is more potent than that in promoting cell proliferation in NSCLC.

Considering gemcitabine mono-therapy is usually administered to elderly patients, to exclude the limitation of age, we recruited NSCLC patients who received routine gemcitabine-platinum chemotherapy (4–6 courses). In parallel, our results demonstrated that platinum has no influence on miR-222-3p expression with or without gemcitabine treatment in advance, which excludes the potential disturbance of platinum, indicating that NSCLC patients with a higher level of circulating exosomic miR-222-3p might experience worse tumor metastasis (*P* = 0.001). Moreover, a higher level of circulating exosomic miR-222-3p correlated with a failed response to gemcitabine therapy (PD), whereas a lower level correlated with an acceptable response (PR).

In summary, our study provides evidence that miR-222-3p was upregulated in both A549-GR–derived exosomes and their donor cells. Upon internalization, these miR-222-3p-rich exosomes induce a more malignant phenotype in recipient sensitive cells by activating the SOCS3/Stat3 signaling pathway. Moreover, circulating exosomic miR-222-3p may function as a biomarker for predicting the response to gemcitabine of patients with advanced NSCLC.

## Conclusions

In conclusion, our study demonstrate the exosomes secreted by GR cells could deliver high levels of miR-222-3p to recipient cells through caveolin- and lipid raft-dependent endocytosis and induce malignant characteristics via targeting SOCS3. The data also suggest that miR-222-3p plays a critical role in gemcitabine resistance and the exosomic miR-222-3p level in serum could serve as a potential prognostic biomarker for predicting gemcitabine sensitivity and NSCLC patients’ response to gemcitabine.

## Additional file

Additional file 1: Figure S1. Expression level of miR-222-3p was detected in A549-P/GR cells and A549-P-KD cells after co-incubation with GR-Exo for 24 h by qRT-PCR. Data represent at least three experiments performed in triplicate. **P* < 0.05; ***P* < 0.01. **Figure S2.** Expression levels of SOCS3 in A549-GR cells after transfection with miR-222-3p mimic and inhibitor were assessed by western blotting. (DOCX 15 kb)

## Abbreviations

GR: Gemcitabine-resistant
miRNA: microRNA
PD: Progressive disease
PR: Partial response
SD: Stable disease
SOCS3: Suppressor of cytokine signaling 3
Stat3: Signal transducer and activator of transcription 3
