# Correction to: Exosomes derived from gemcitabine resistant cells transfer malignant phenotypic traits via delivery of miRNA-222-3p

**DOI:** 10.1186/s12943-021-01320-y

**Published:** 2021-02-17

**Authors:** Feng Wei, Chengyuan Ma, Tong Zhou, Xuechao Dong, Qinghua Luo, Li Geng, Lijuan Ding, Yandong Zhang, Li Zhang, Nan Li, Yang Li, Yan Liu

**Affiliations:** 1 Department of Hepatobiliary & Pancreas, The First Hospital of Jilin University, Changchun, 130021 Jilin China; 2 Genetic Engineering Laboratory of PLA, The Eleventh Institute of Academy of Military Medical Sciences of PLA, Changchun, 130122 Jilin China; 3 Department of Respiratory Medicine, The First Hospital of Jilin University, Changchun, 130021 Jilin China; 4 Department of neurosurgery, The First Hospital of Jilin University, Changchun, 130021 Jilin China; 5 Department of Endocrinology, The First Hospital of Jilin University, Changchun, 130021 Jilin China; 6 Department of General Surgery, The Second Hospital of Jilin University, Changchun, 130041 Jilin China; 7 College of Veterinary Medicine, Yangzhou University, Yangzhou, 225009 Jiangsu China

**Correction to: Mol Cancer 16, 132 (2017)**

**https://doi.org/10.1186/s12943-017-0694-8**

Following the publication of the original article [1], the authors reported that some mistakes were made while previously arranging the images into Figs. 1F, 2H, and 4C/D during submission of the manuscript which looks like an image duplication.

**Fig. 1:**
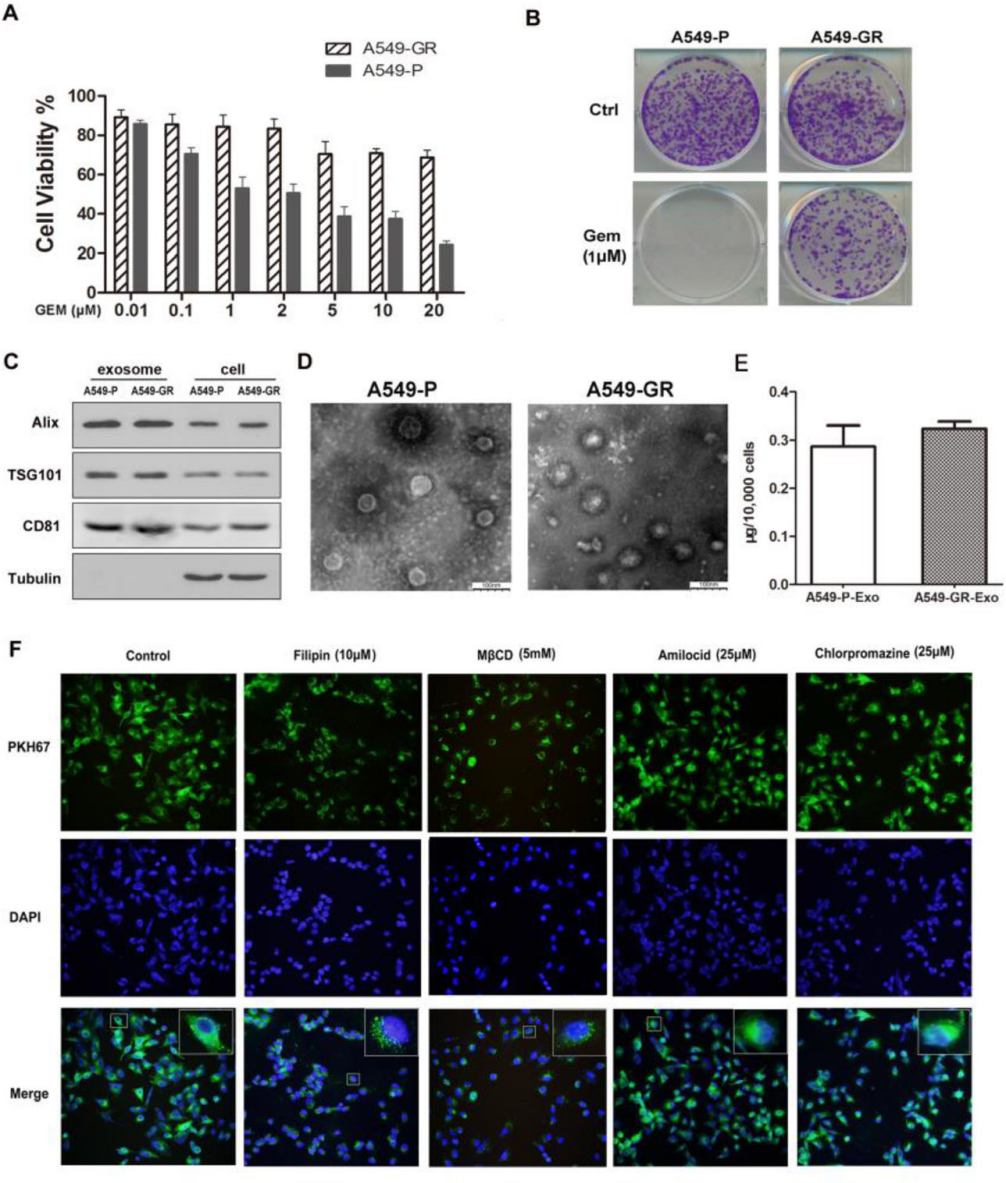
Gemcitabine-resistant cells shed exosomes that readily enter A549-P cells. **a** A549-P/GR cells were treated with increasing concentrations of gemcitabine for 72 h. Cell growth was analyzed by MTS assay. Data represent at least three experiments performed in triplicate. Error bars represent ± SD. *P < 0.01; **P < 0.001. **b** A549-P/GR cells were treated with gemcitabine (1 μM) and examined by colony formation assay. **c** The presence of TSG101, CD81, and Alix in 15 μg of exosomes was analyzed by western blot. **d** Exosomes were observed by transmission electron microscopy. Scale bar, 100 μm. **e** Quantification of exosomes expelled from equal numbers of A549-P/GR cells by BCA analysis. **f** A549-P cells were treated with different pharmacological inhibitors of endocytic pathways for 2 h. A549-GR–derived exosomes (GR-Exo) were dyed with PKH67 (green) and incubated with pretreated cells for 4 h, and exosome uptake was viewed under confocal microscopy

**Fig. 2:**
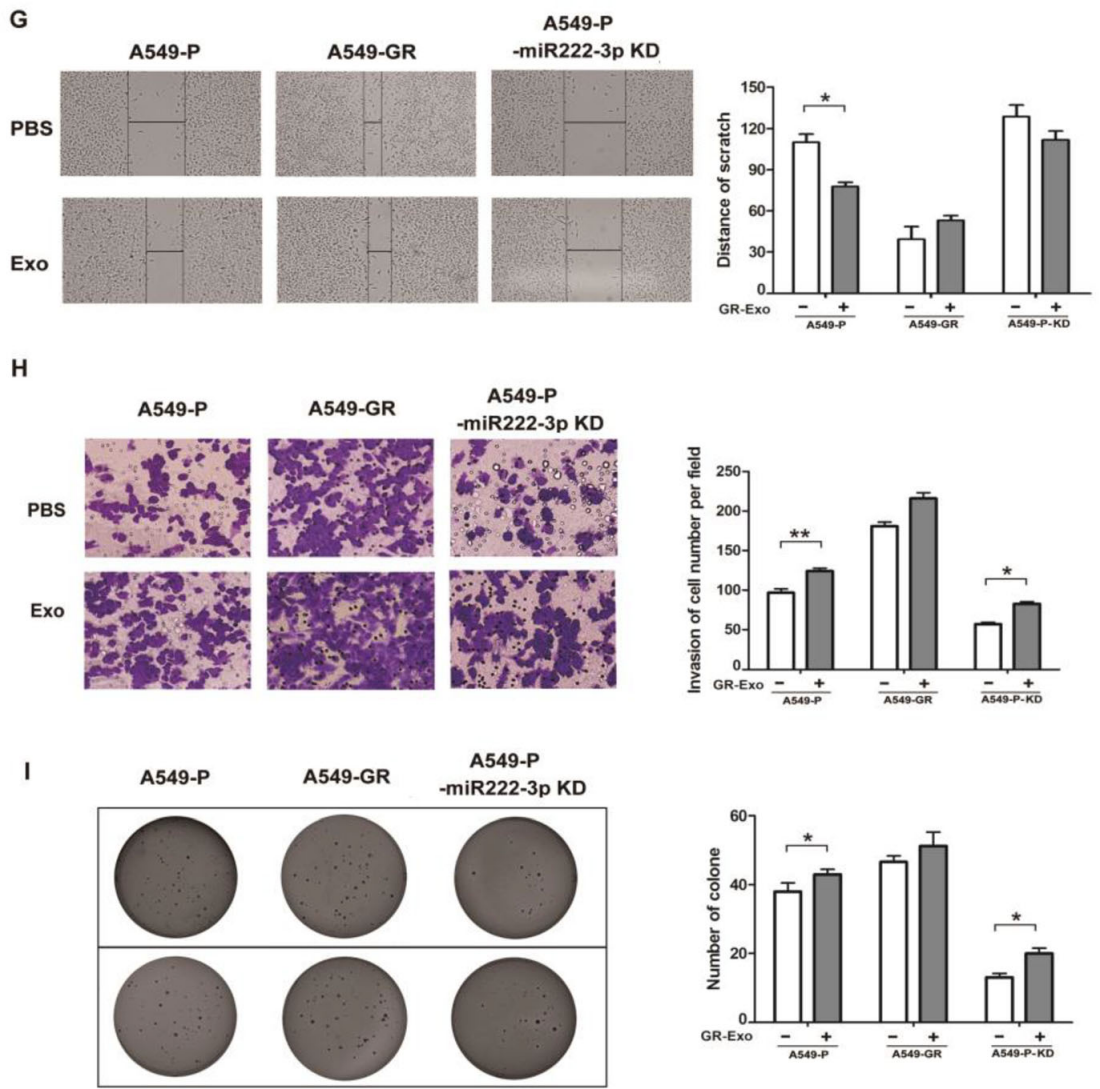
GR-Exo deliver miRNA-222-3p into recipient cells, enhance gemcitabine resistance, promote cell metastasis and proliferation. **a** Heatmap of differential miRNA expression between A549-GR and A549-P exosomes. Gene expression data were obtained using a human microRNA array. Expression values shown are mean centered. Red: increased expression, Green: decreased expression. **b** miR-222-3p expression was detected in A549-P/GR cells and A549-P/GR–derived exosomes by qRT-PCR. **c** miR-222-3p expression was detected in A549-P cells after co-incubation with P-Exo or GR-Exo for 24 h by qRT-PCR. **d** miR-222-3p expression was detected in H460 and H157 cells after co-incubation with P-Exo or GR-Exo by qRT-PCR. **e** The growth rates of A549-P and A549-P-KD cells in the absence or presence of GR-Exo were assessed using MTS assay. **f** Cell growth was assessed by MTS after treatment with gemcitabine for 72 h in the absence or presence of P-Exo or GR-Exo. **g** The migration of exosome-treated cells was assessed using wound-healing assay. Right, quantitative analysis of invasive cells. **h** Transwell assay was performed to assess cell invasion. Right, quantitative analysis of scratch wound closure. **i** Soft agar assay was performed to examine the anchorage-independent survival of cells. Right, quantitative analysis of cell clones. Data represent at least three experiments performed in triplicate. *P < 0.05; **P < 0.01

**Fig. 4:**
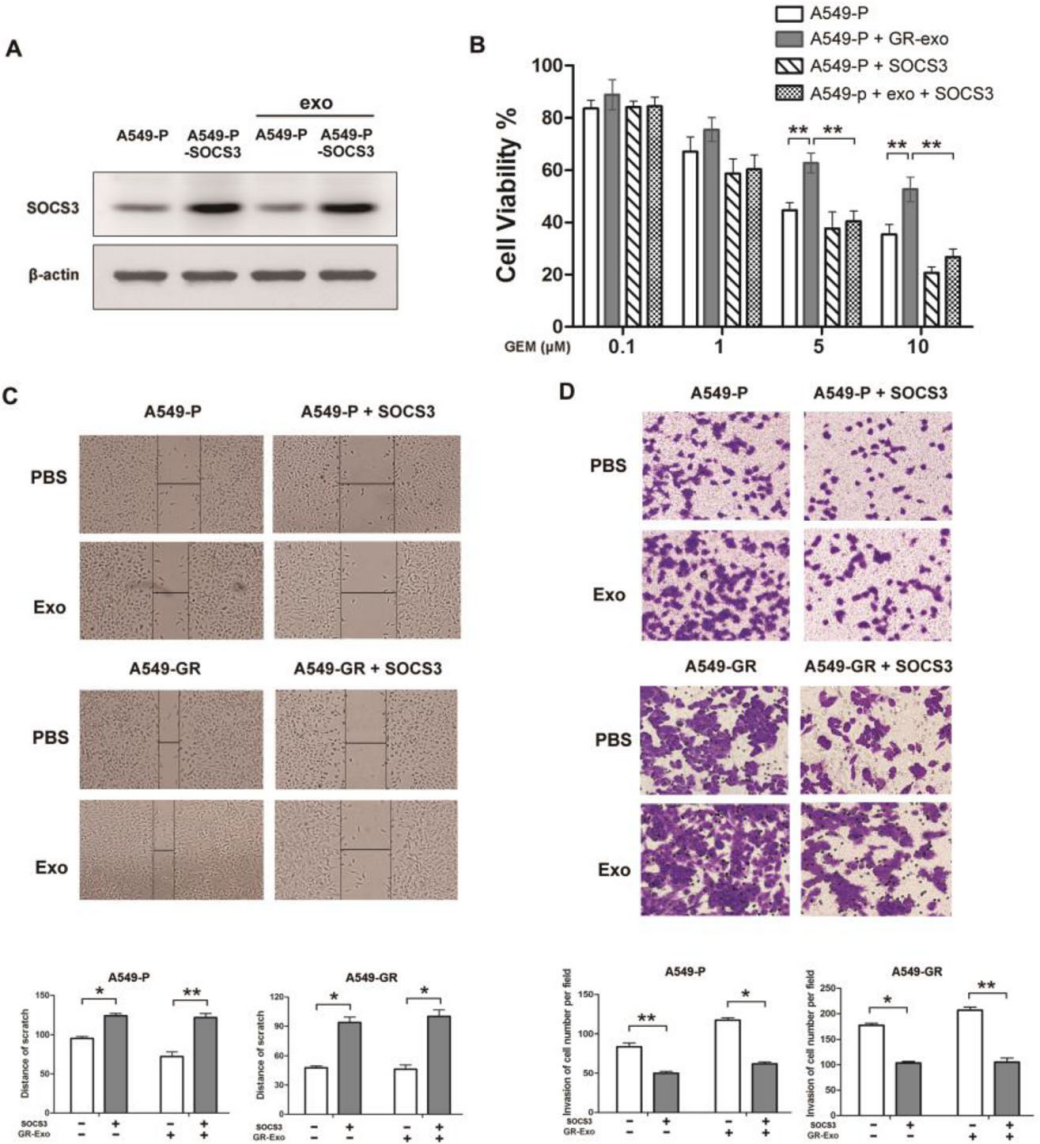
Exogenous overexpression of SOCS3 rescues A549-P cells from GR-Exo–induced stronger malignancy. A549-P or A549-GR cells were transfected with GV-144-SOCS3 plasmid and treated with GR-Exo. **a** SOCS3 expression was assessed by western blotting. **b** Cell viability was assessed by MTS after co-treatment with gemcitabine for 72 h. Data are representative of three experiments. Error bars represent ±SD. *P < 0.05; **P < 0.01 vs. control. **c** Wound-healing assay and **d** Transwell assay were performed to assess cell migration and invasion. Lower, quantitative analysis of scratch wound closure (**c**) or cell invasion (**d**). Data represented at least three experiments performed in triplicate. *P < 0.05; **P < 0.01

The histogram does not need to be changed, because we did not use the wrong picture when analyzing the original data.

After evaluating the influence of this negligence on the conclusions of the manuscript, we believe that this negligence does not alter the conclusions drawn in the manuscript. Nonetheless, we apologize for any confusion these original images may have caused.

The correct figures are updated below.

## References

[1] Wei F, Ma C, Zhou T (2017). Exosomes derived from gemcitabine-resistant cells transfer malignant phenotypic traits via delivery of miRNA-222-3p. Mol Cancer.

