# Supplementary material for: Exosomes derived from gemcitabine-resistant cells transfer malignant phenotypic traits via delivery of miRNA-222-3p
Source: Mol Cancer. 2017 Jul 25;16:132. doi: 10.1186/s12943-017-0694-8 (PMC5526308; doi:10.1186/s12943-017-0694-8)
Supplement: Additional file 1: Figure S1. — Expression level of miR-222-3p was detected in A549-P/GR cells and A549-P-KD cells after co-incubation with GR-Exo for 24 h by qRT-PCR. Data represent at least three experiments performed in triplicate. *P < 0.05; **P < 0.01. Figure S2. Expression levels of SOCS3 in A549-GR cells after transfection with miR-222-3p mimic and inhibitor were assessed by western blotting. (DOCX 15 kb) [file 12943_2017_694_MOESM1_ESM.docx]

**Supplemental figure legends**

**Fig. S1** Expression level of miR-222-3p was detected in A549-P/GR cells and A549-P-KD cells after co-incubation with GR-Exo for 24 h by qRT-PCR. Data represent at least three experiments performed in triplicate. **P*<0.05; ***P*<0.01.

**Fig. S2** Expression levels of SOCS3 in A549-GR cells after transfection with miR-222-3p mimic and inhibitor were assessed by western blotting.
